# Supplementary material for: Influence of Ionomer and Cyanuric Acid on Antistatic, Mechanical, Thermal, and Rheological Properties of Extruded Carbon Nanotube (CNT)/Polyoxymethylene (POM) Nanocomposites
Source: Polymers (Basel). 2022 Apr 30;14(9):1849. doi: 10.3390/polym14091849 (PMC9102448; doi:10.3390/polym14091849)
Supplement: Supplementary file 1 [file polymers-14-01849-s001.zip › polymers-1678774-supplementary.pdf]

Supplementary information

# Influence of Ionomer and Cyanuric Acid on Antistatic, Mechanical, Thermal, and Rheological Properties of Extruded Carbon Nanotube (CNT)/Polyoxymethylene (POM) Nanocomposites

**Sang-Seok Yun [1,†], Dong-Hyuk Shin [2,†] and Keon-Soo Jang [1,*]**

1 Department of Polymer Engineering, School of Chemical and Materials Engineering, The University of Suwon, Hwaseong 18323, Gyeonggi-do, Korea; fourdai@suwon.ac.kr
2 Woosung Chemical Co., 150, Pungsesandan-ro, Pungse-myeon, Dongnam-gu, Cheonan-si 31214, Chungcheongnam-do, Korea; sdh@metapoly.co.kr
* Correspondence: ksjang@suwon.ac.kr or ksjang4444@gmail.com
† These authors contributed equally to this work.

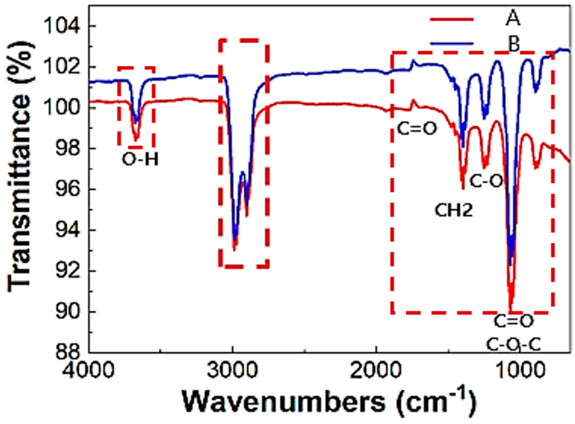


**Figure S1.** FTIR spectra of CNTs: (A) upper and (b) bottom parts of 1 kg CNT package.

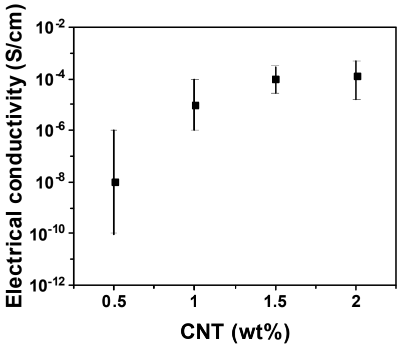


**Figure S2.** Electrical conductivities of POM/CNT nanocomposites as a function of CNT loading.

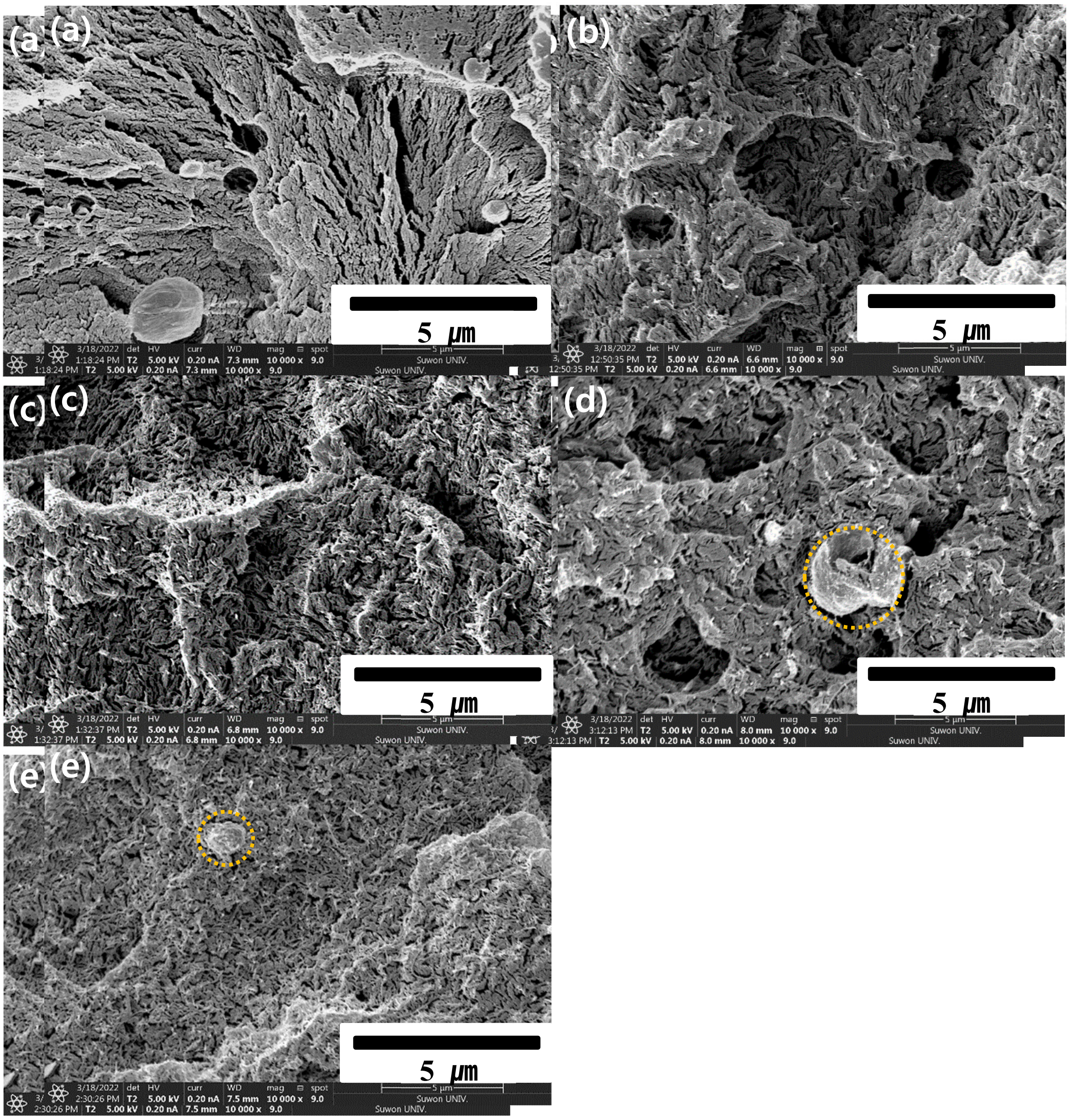


**Figure S3.** SEM images of neat POM and POM/CNT nanocomposites with a magnification of × 10,000: (a) pristine POM, (b) POM/C0.5, (c) POM/C1, (d) POM/C1.5, and (e) POM/C2.

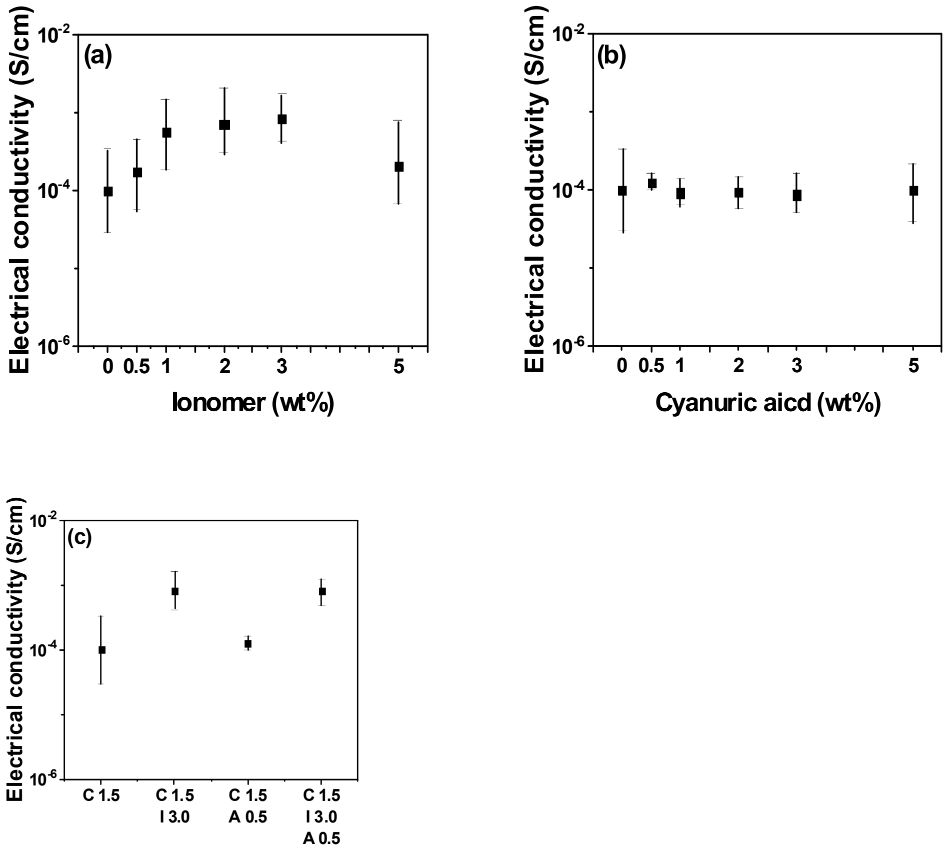


**Figure S4.** Electrical conductivities of POM/C1.5/I, POM/C1.5/A and various nanocomposites (POM/C1.5, POM/C1.5/I3, POM/C1.5/A0.5, and POM/C1.5/I3/A0.5) nanocomposites: (a) POM/C1.5/I, (b) POM/C1.5/A, (c) POM/C1.5, POM/C1.5/I3, POM/C1.5/A0.5, and POM/C1.5/I3/A0.5.

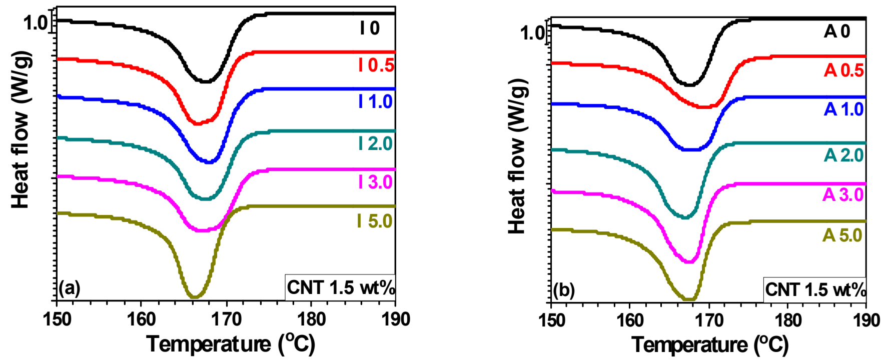


**Figure S5.** DSC heating scans of pristine POM/C, POM/C/I, and POM/C/A nanocomposites with different additive contents: (a) Ionomer, and (b) Cyanuric acid.

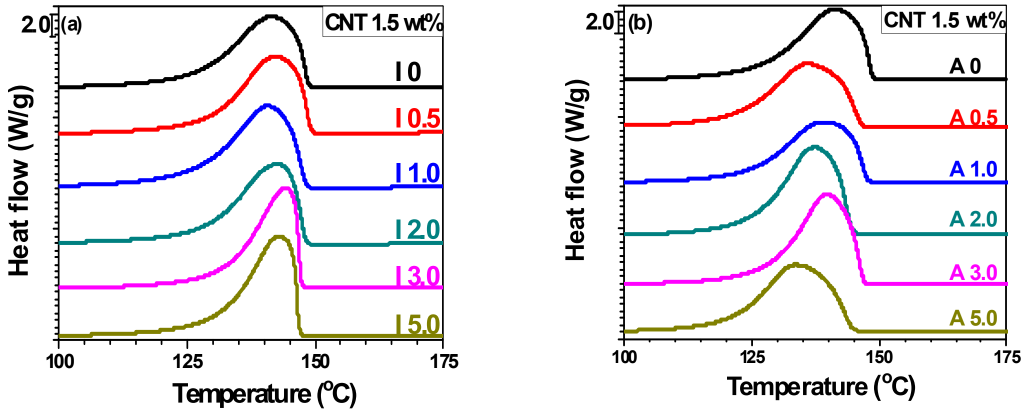


**Figure S6.** DSC cooling scans of pristine POM/C, POM/C/I, and POM/C/A nanocomposites with different additive contents: (a) Ionomer and (b) Cyanuric acid.

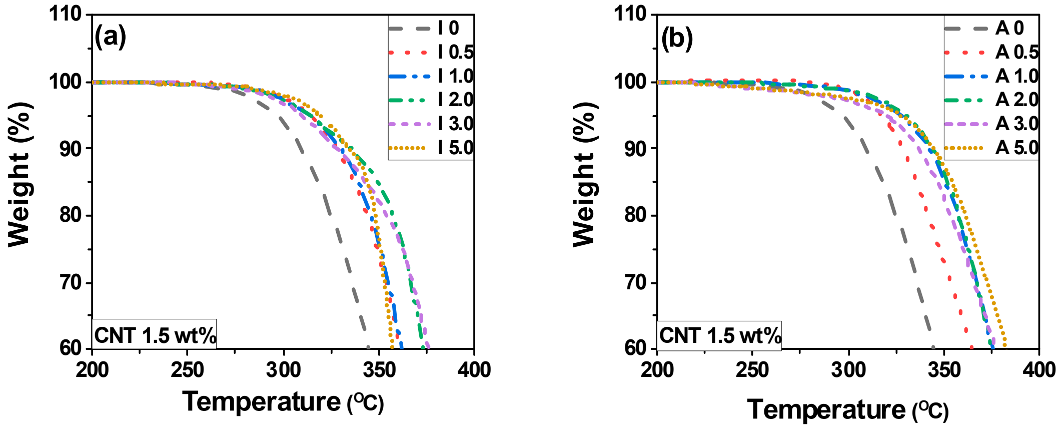


**Figure S7.** TGA curves of pristine POM/C, POM/C/I, and POM/C/A nanocomposites with different additive contents: (a) Ionomer and (b) Cyanuric acid.

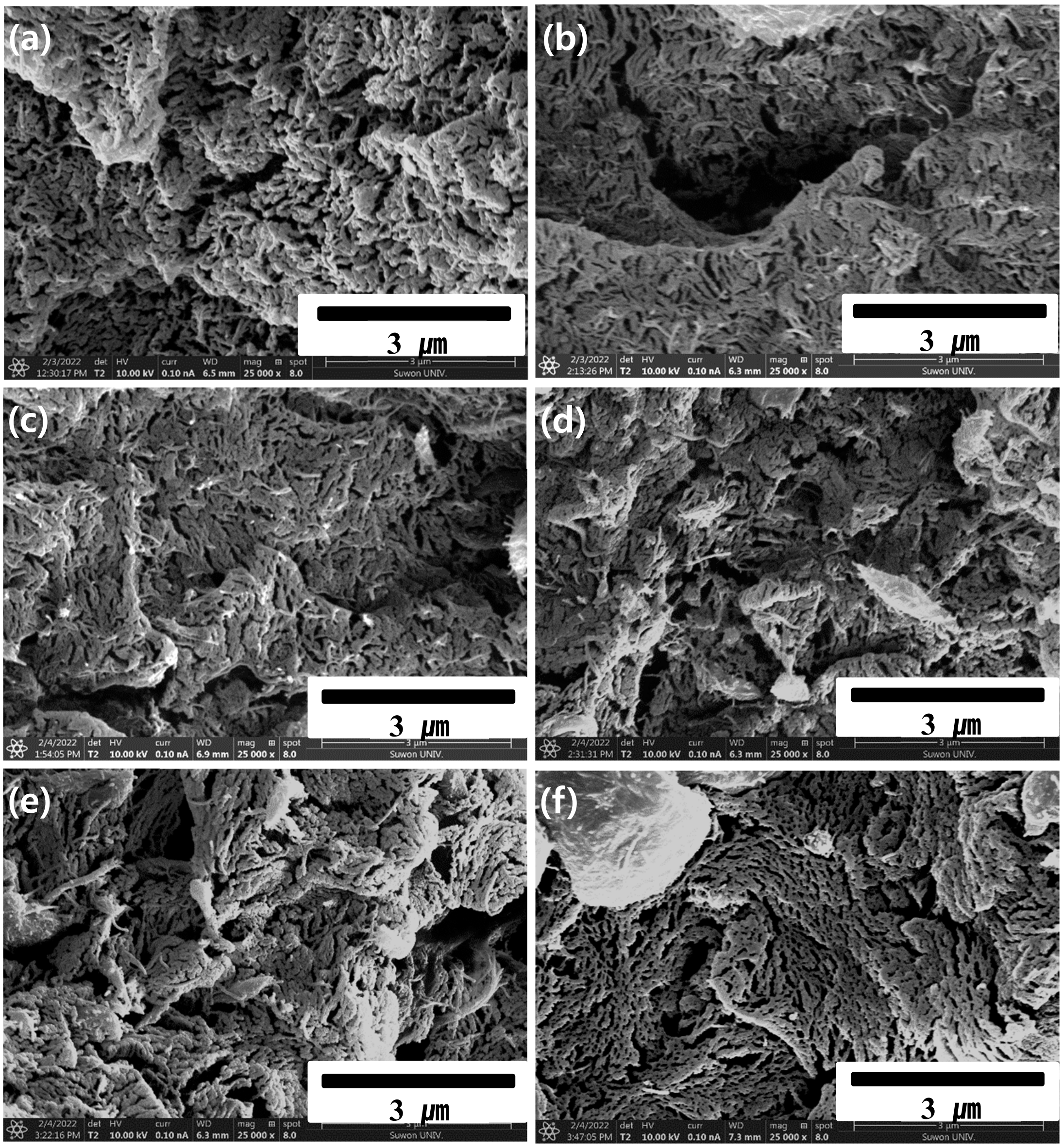


**Figure S8.** SEM images of POM/CNT (POM/C1.5) and POM/CNT/ionomer (POM/C1.5/I) nanocomposites as a function of ionomer with a magnification of × 25,000: (a) I0, (b) I0.5, (c) I1, (d) I2, (e) I3, and (f) I5.

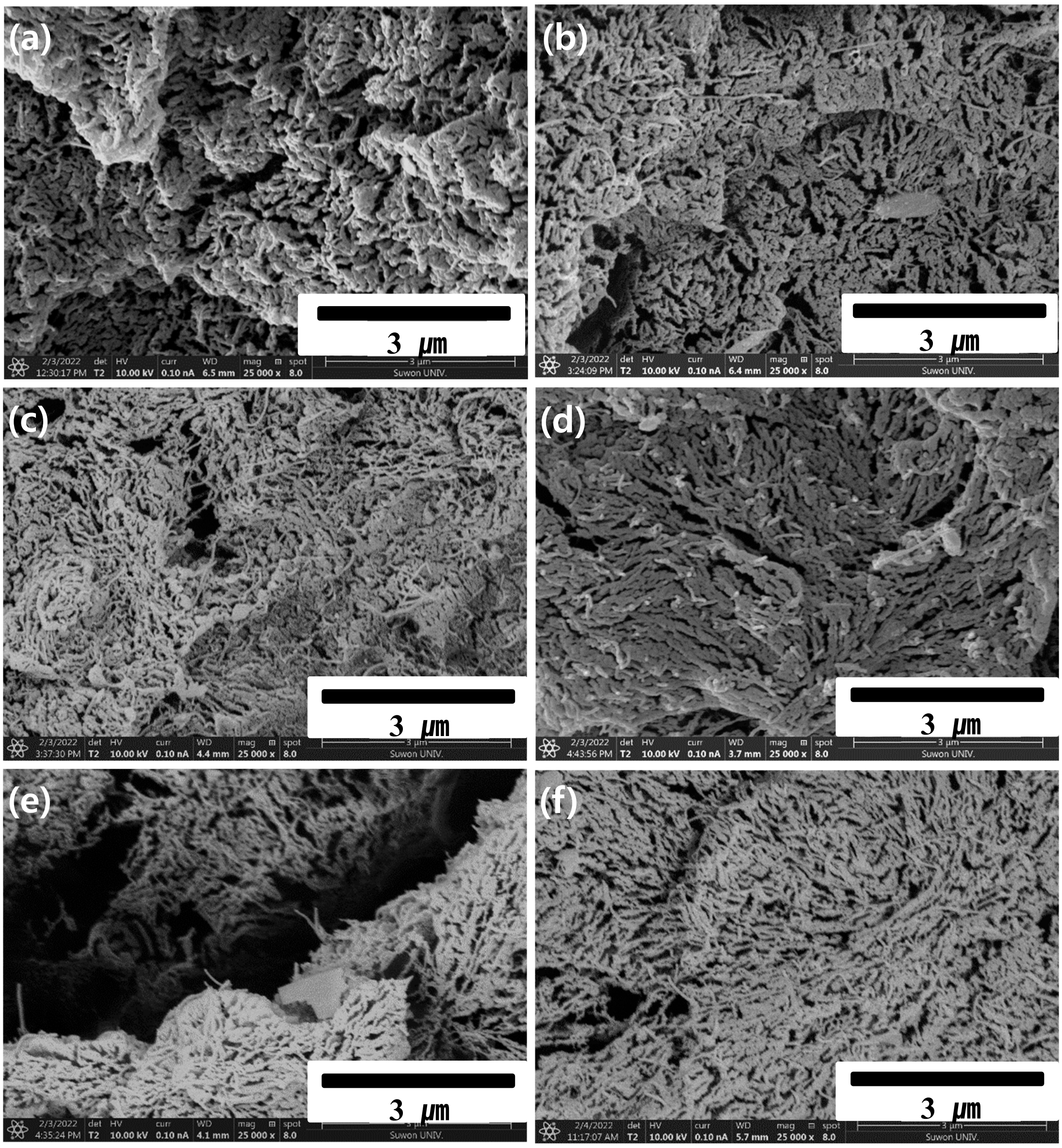


**Figure S9.** SEM images of POM/CNT (POM/C1.5) and POM/CNT/cyanuric acid (POM/C1.5/A) nanocomposites as a function of cyanuric acid with a magnification of × 25,000: (a) A0, (b) A0.5, (c) A1, (d) A2, (e) A3, and (f) A5.

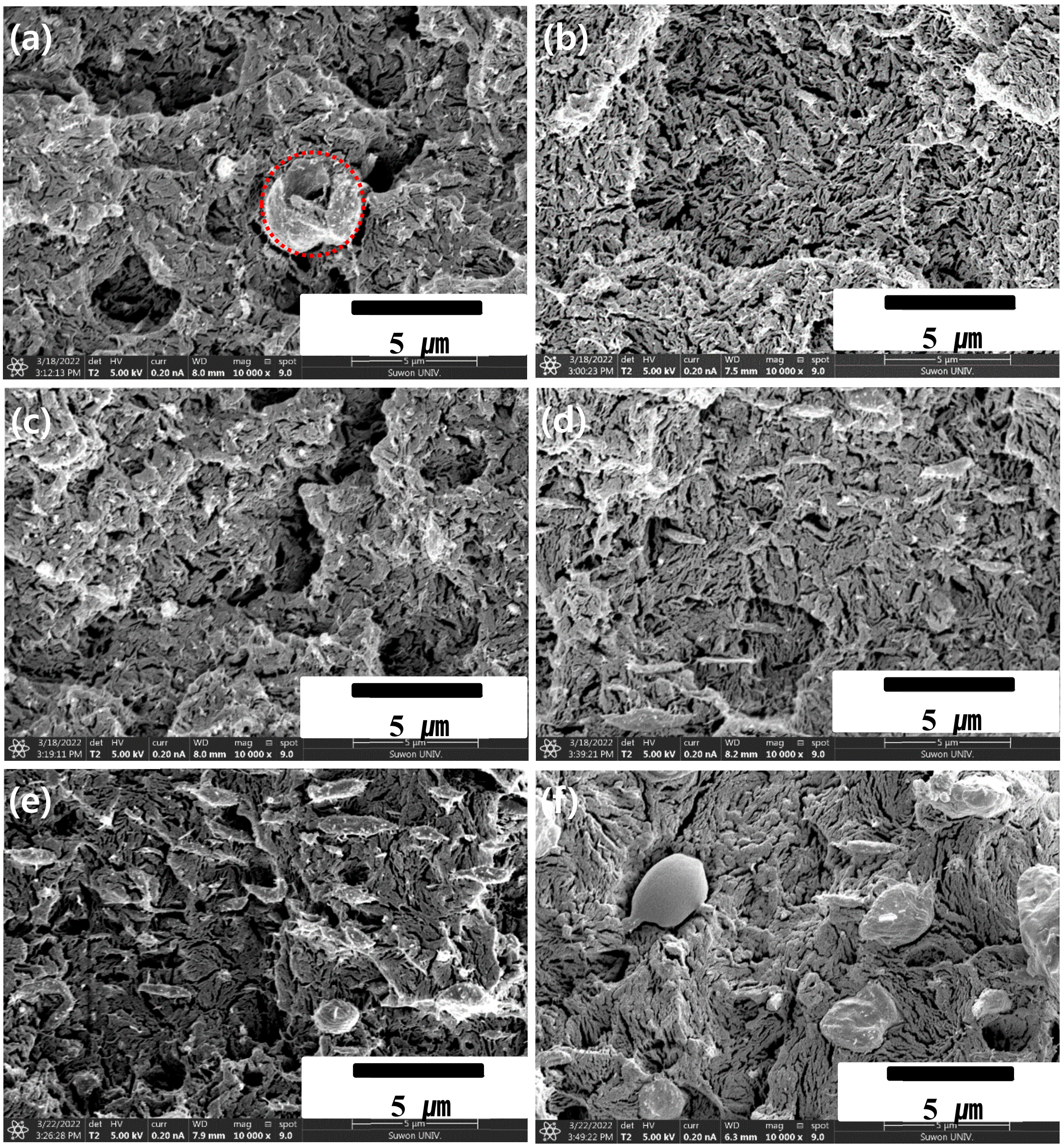


**Figure S10.** SEM images of POM/CNT (POM/C1.5) and POM/CNT/ionomer (POM/C1.5/I) nanocomposites as a function of ionomer with a magnification of × 10,000: (a) I0, (b) I0.5, (c) I1, (d) I2, (e) I3, and (f) I5.

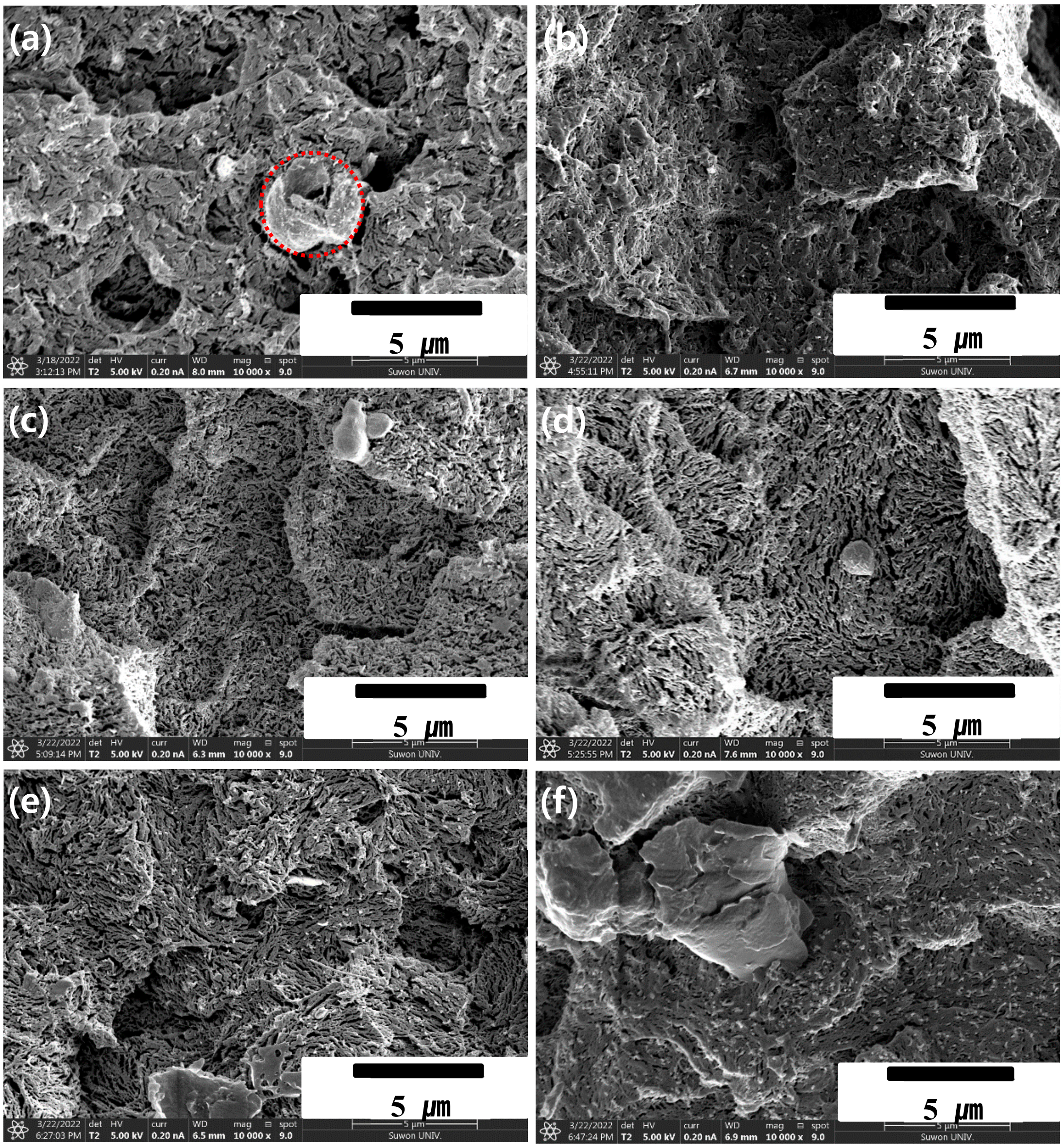


**Figure S11.** SEM images of POM/CNT (POM/C1.5) and POM/CNT/cyanuric acid (POM/C1.5/M) nanocomposites as a function of cyanuric acid with a magnification of × 10,000: (a) A0, (b) A0.5, (c) A1, (d) A2, (e) A3, and (f) A5.

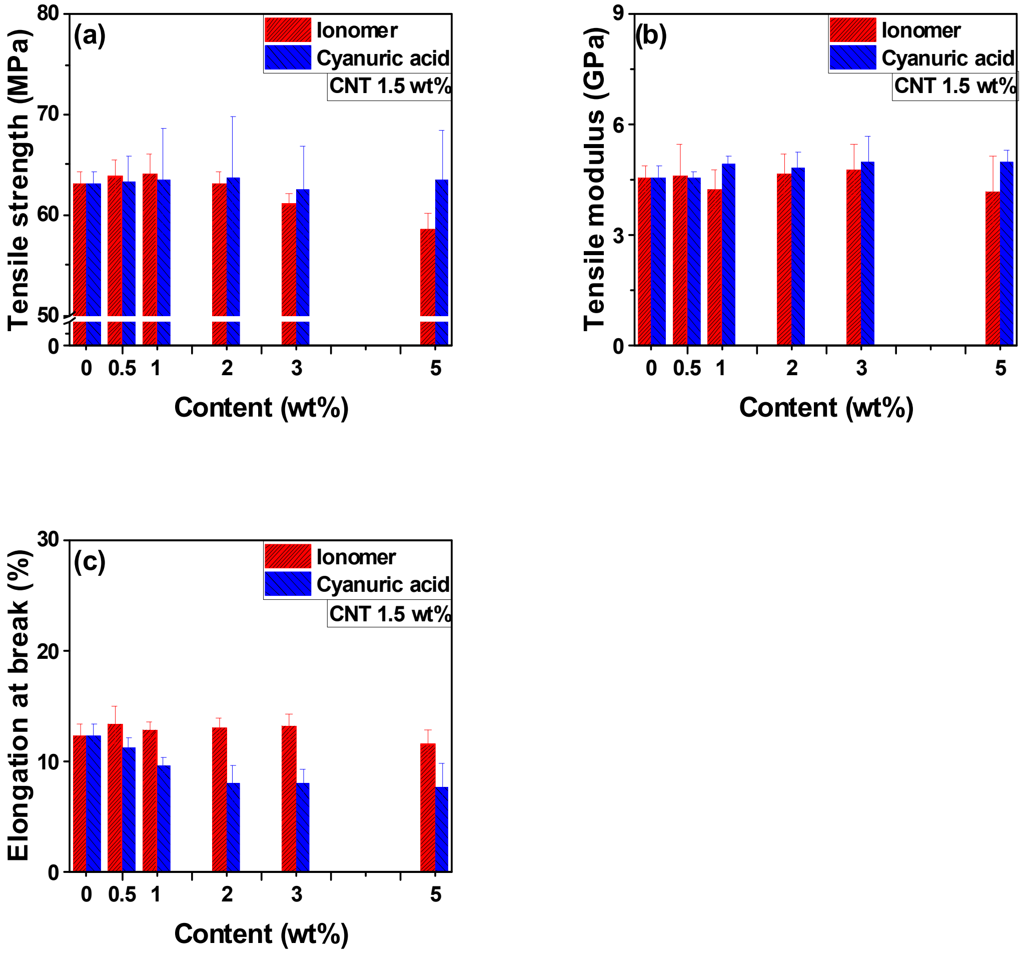


**Figure S12.** Tensile properties of POM/C, POM/C/I, and POM/C/A nanocomposites: (a) tensile strength, (b) tensile modulus, and (c) elongation at break.

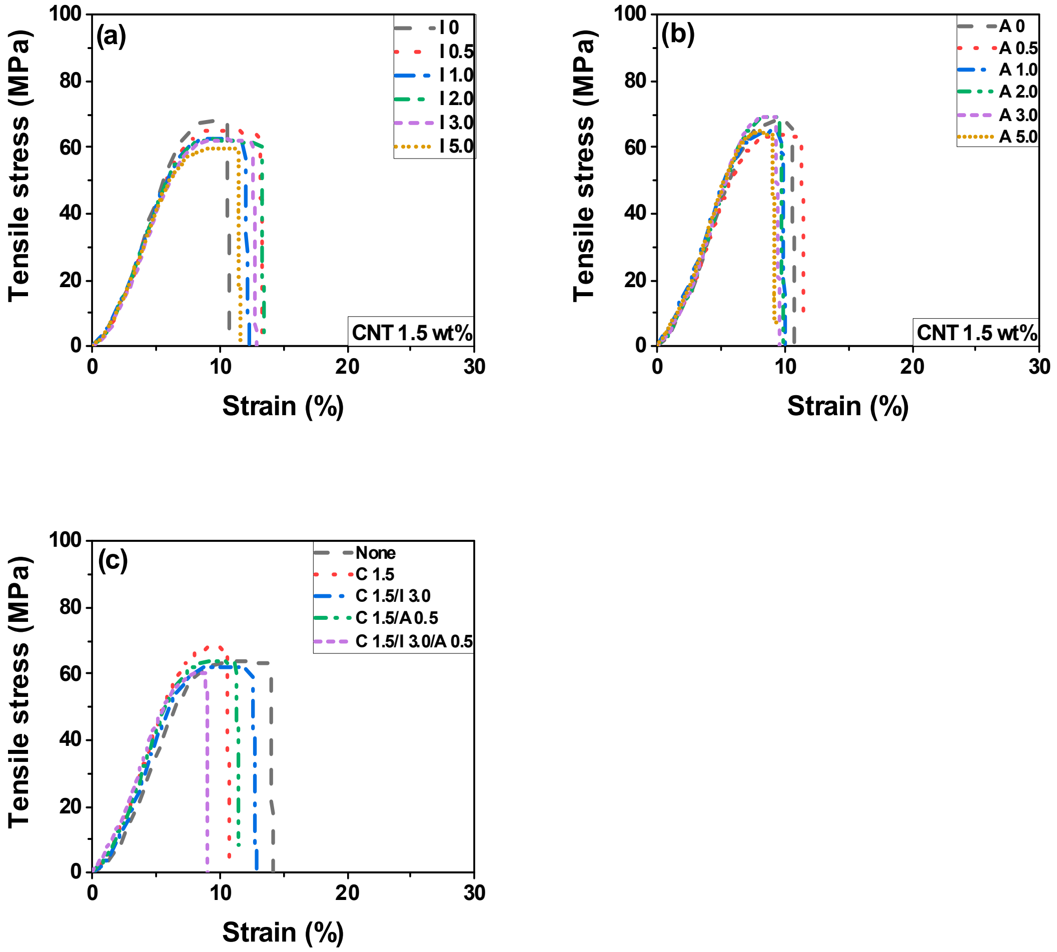


**Figure S13.** Tensile stress-strain of POM/C, POM/C/I, and POM/C/A nanocomposites with different additive concentrations: (a) Ionomer, (b) Cyanuric acid, and (c) combination of ionomer/cyanuric acid.

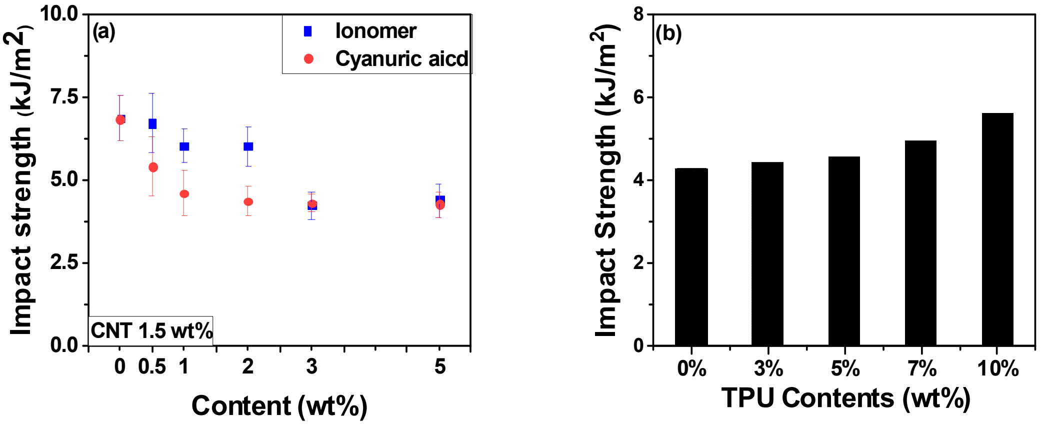


**Figure S14.** Izod impact strengths of POM/C1.5, POM/C1.5/I, POM/C1.5/A, and POM/C1.5/I3/A0.5/TPU nanocomposites as a function of additive loading (a) POM/C1.5, POM/C1.5/I, and POM/C1.5/A and (b) POM/C1.5/I3/A0.5/TPU

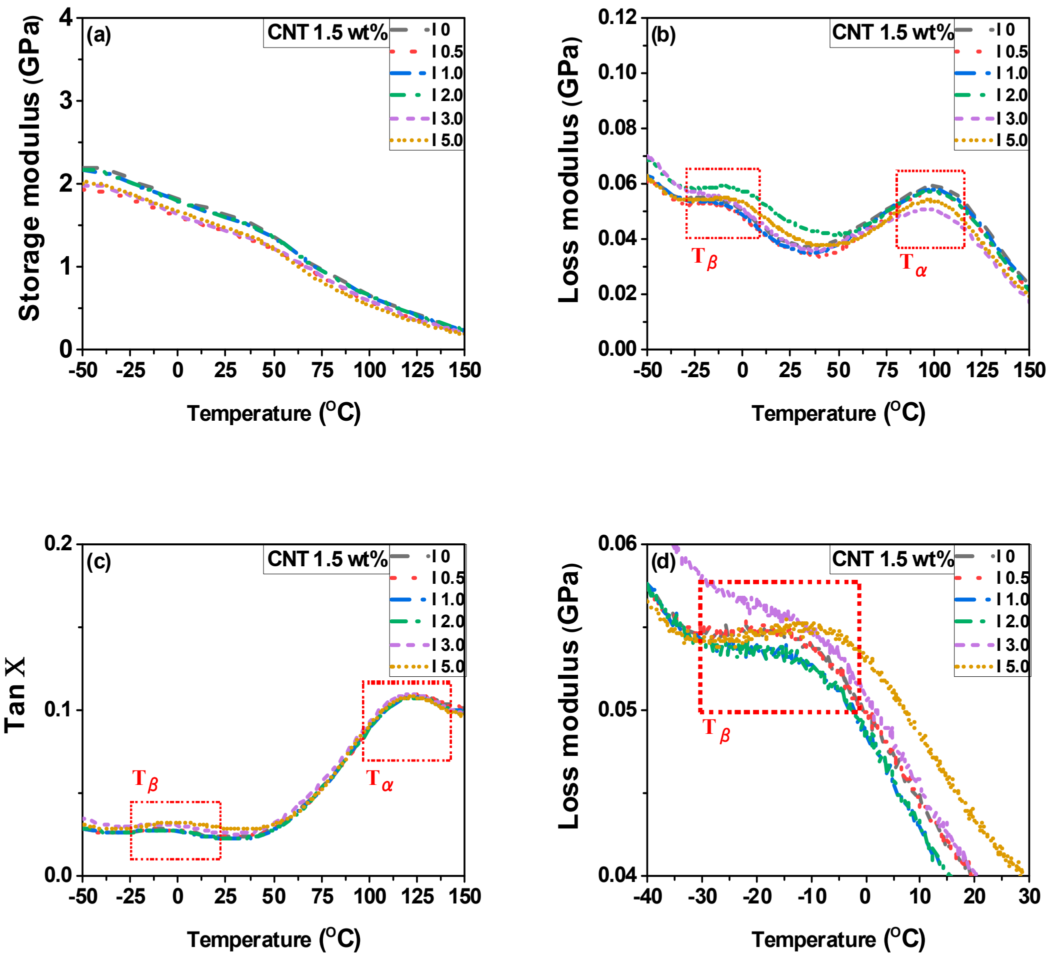

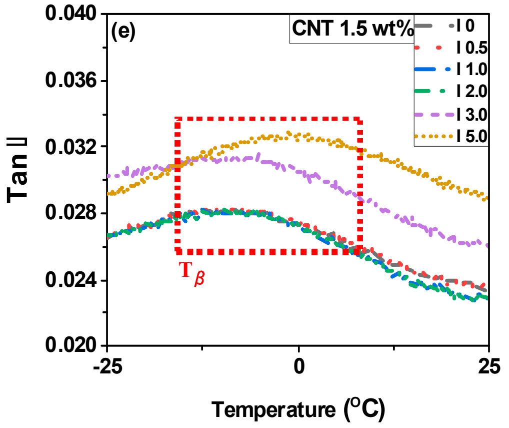


**Figure S15.** DMA data of POM/C1.5 and POM/C1.5/I nanocomposites with different ionomer concentrations: (a) Storage modulus, (b) loss modulus, and (c) tan δ.

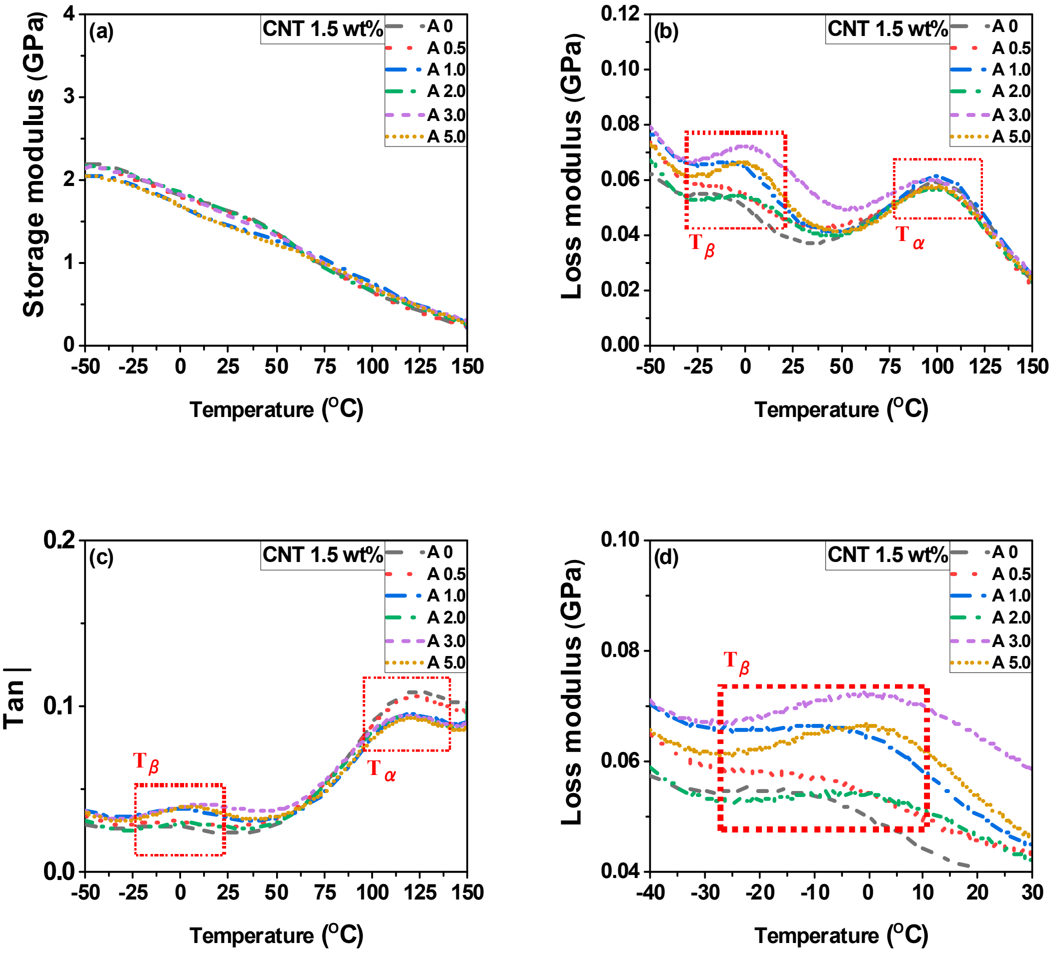

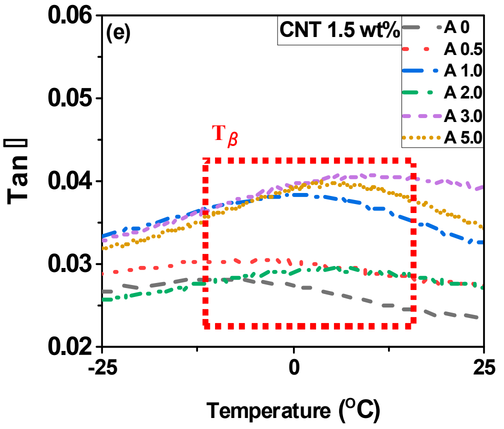


**Figure S16.** DMA data of POM/C1.5 and POM/C1.5/A nanocomposites with different cyanuric acid concentrations: (a) Storage modulus, (b,d) loss modulus, and (c,e) tan δ.

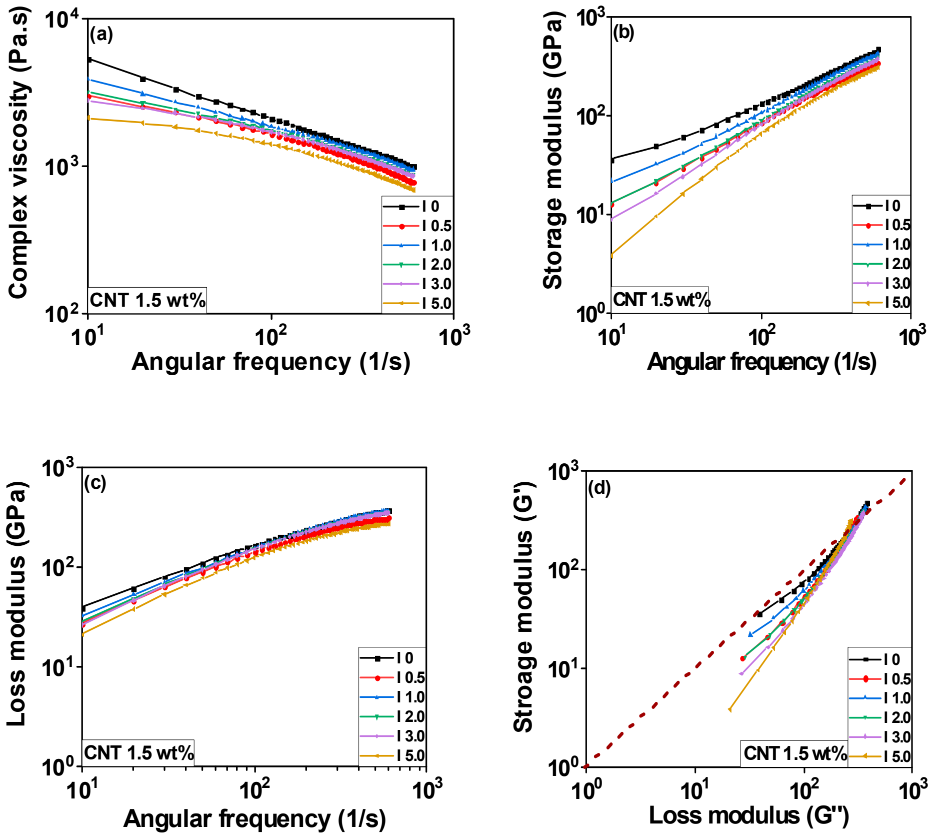


**Figure S17.** Rheological properties of the POM/C1.5 and POM/C1.5/I nanocomposites: (a) Complex viscosity, (b) shear storage modulus (*G'*), (c) shear loss modulus (*G"*) vs. frequency, and (d) *G'* vs. *G"*

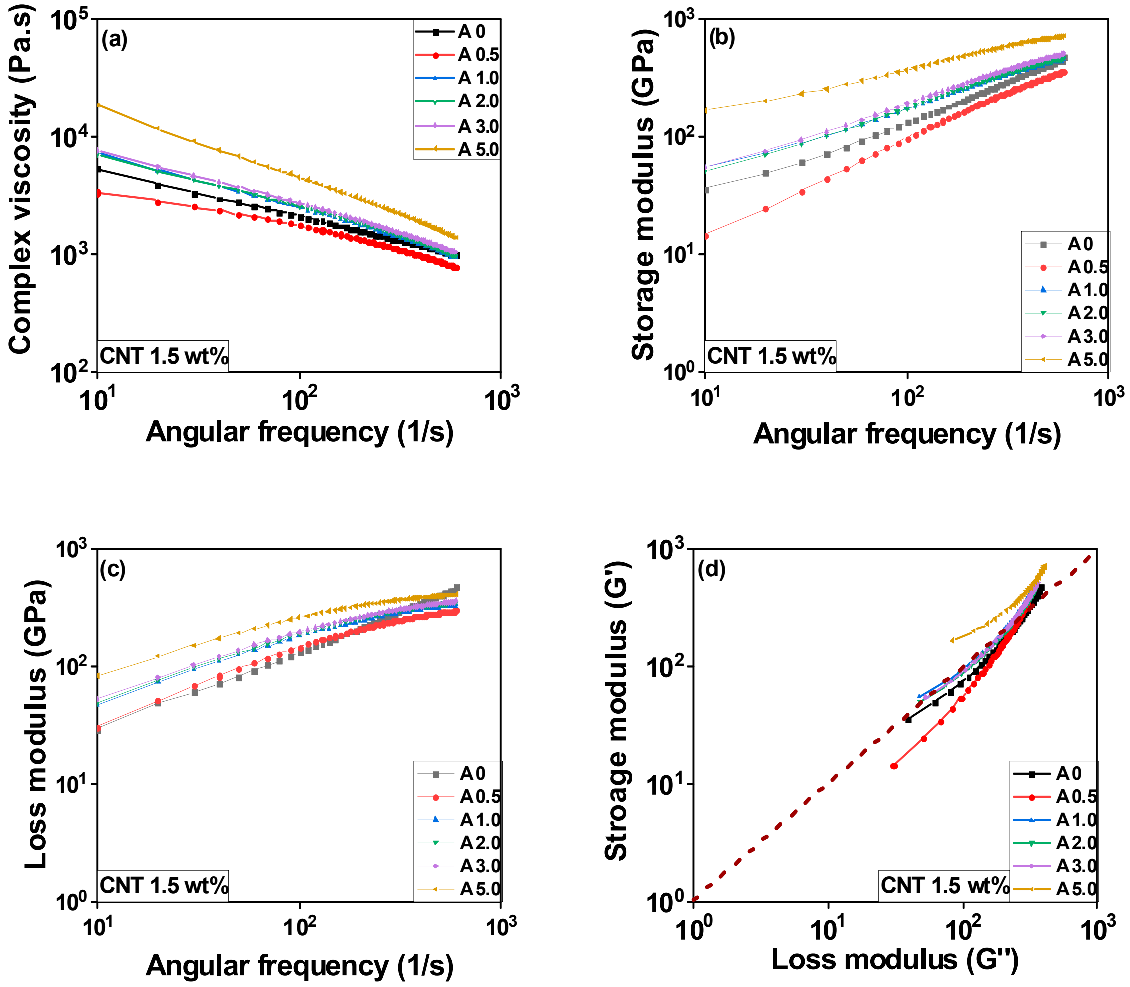


**Figure S18.** Rheological properties of the POM/C1.5 and POM/C1.5/A nanocomposites: (a) Complex viscosity, (b) shear storage modulus (*G'*), (c) shear loss modulus (*G''*) vs. frequency, and (d) *G'* vs. *G''*

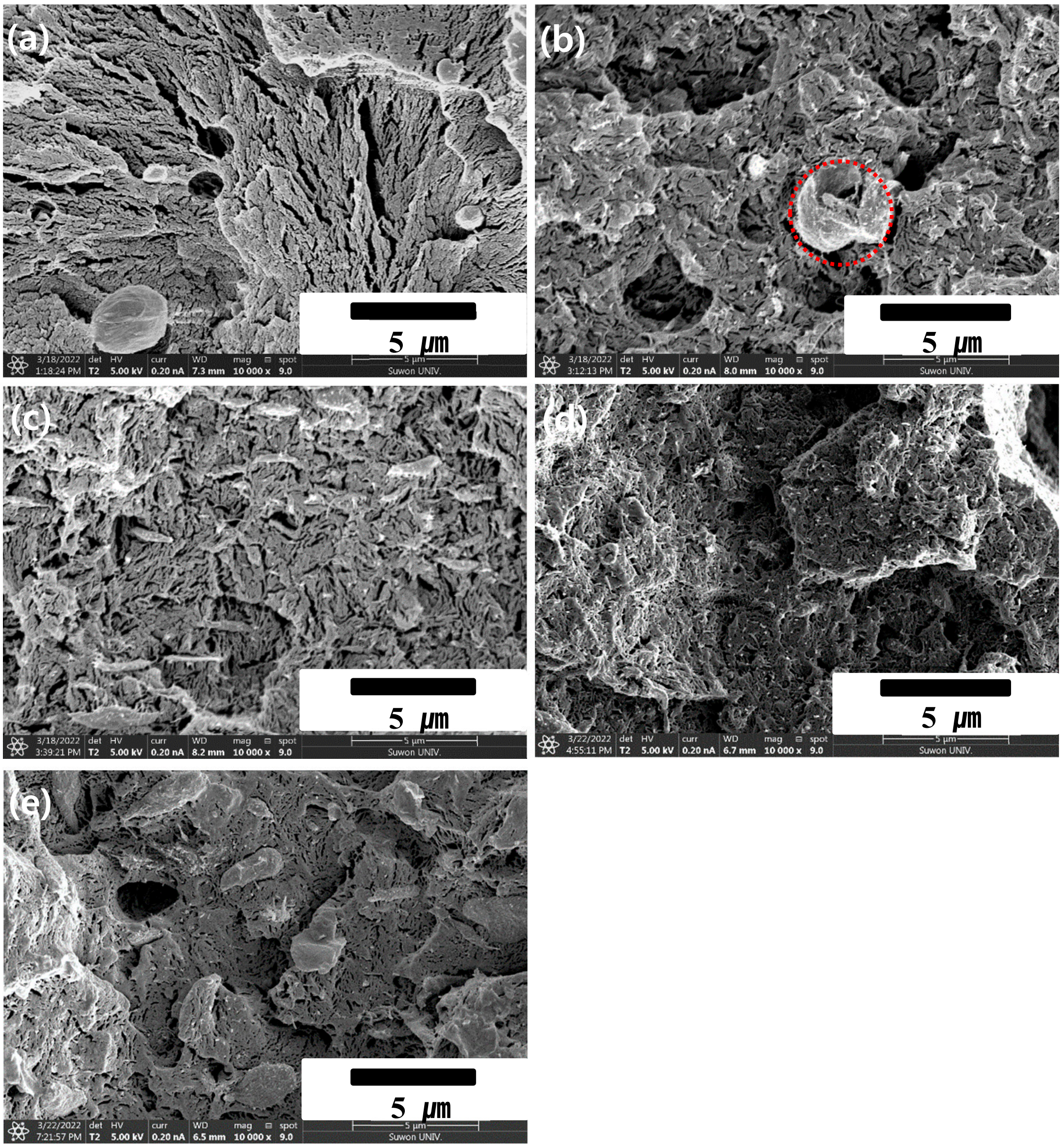


**Figure S19.** SEM images of pristine POM, POM/CNT, POM/C1.5/I3, POM/C1.5/A0.5, and POM/C1.5/I3/A0.5 nanocomposites with a magnification of × 10,000: (a) None, (b) C 1.5, (c) C 1.5/I 3.0 , (d) C 1.5/A 0.5, and (e) C 1.5/I 3.0/A 0.5

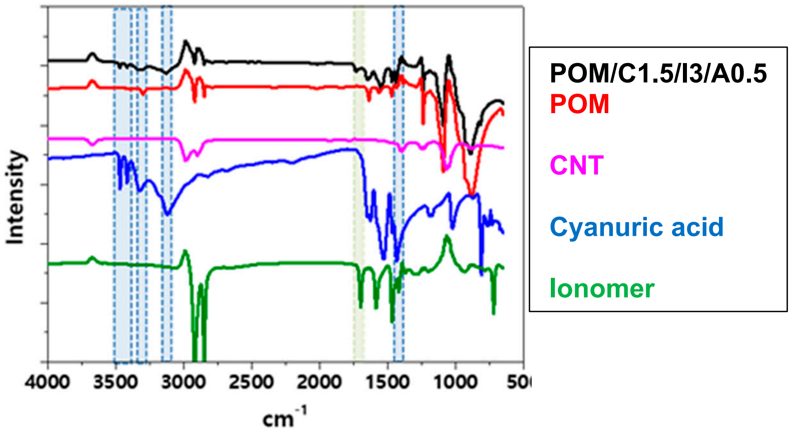


**Figure S20.** FT-IR spectra of POM, CNT, ionomer, cyanuric acid, and nanocomposite (POM/C1.5/I3/A0.5).
